# Lessons from ten years of genome-wide association studies of asthma

**DOI:** 10.1038/cti.2017.54

**Published:** 2017-12-15

**Authors:** Cristina T Vicente, Joana A Revez, Manuel A R Ferreira

**Affiliations:** 1 QIMR Berghofer Medical Research Institute, Brisbane, QLD, Australia

## Abstract

Twenty-five genome-wide association studies (GWAS) of asthma were published between 2007 and 2016, the largest with a sample size of 157242 individuals. Across these studies, 39 genetic variants in low linkage disequilibrium (LD) with each other were reported to associate with disease risk at a significance threshold of *P*<5 × 10^−8^, including 31 in populations of European ancestry. Results from analyses of the UK Biobank data (*n*=380 503) indicate that at least 28 of the 31 associations reported in Europeans represent true-positive findings, collectively explaining 2.5% of the variation in disease liability (median of 0.06% per variant). We identified 49 transcripts as likely target genes of the published asthma risk variants, mostly based on LD with expression quantitative trait loci (eQTL). Of these genes, 16 were previously implicated in disease pathophysiology by functional studies, including *TSLP*, *TNFSF4*, *ADORA1*, *CHIT1* and *USF1*. In contrast, at present, there is limited or no functional evidence directly implicating the remaining 33 likely target genes in asthma pathophysiology. Some of these genes have a known function that is relevant to allergic disease, including *F11R, CD247, PGAP3, AAGAB*, *CAMK4* and *PEX14*, and so could be prioritized for functional follow-up. We conclude by highlighting three areas of research that are essential to help translate GWAS findings into clinical research or practice, namely validation of target gene predictions, understanding target gene function and their role in disease pathophysiology and genomics-guided prioritization of targets for drug development.

## Introduction

Asthma is a common and chronic inflammatory disease of the airways, specifically affecting the bronchi and bronchioli. Bronchial inflammation, which results in airway narrowing and shortness of breath symptoms, is generally caused by innate and adaptive immune responses to inhaled viruses and/or allergens.^1^ These and other environmental exposures are strong risk factors for disease onset and exacerbations but, based on twin studies, account for less than half of the overall disease liability.^2, 3, 4^ The remaining disease risk is largely explained by inherited genetic factors, with gene-by-environment interaction effects also thought to play a role. Given this high heritability, there has been a long-standing interest in identifying specific genetic risk factors for asthma, initially through linkage analysis (1989^5^ to 2010^6^) and subsequently through candidate-gene association studies (since 1995^7^). Linkage studies were largely (if not entirely) unsuccessful because this approach is only adequately powered with realistic sample sizes to identify very large genetic effects,^8^ which we now know do not exist for asthma. On the other hand, most published candidate-gene studies suffered from a number of methodological limitations (for example, small number of samples and genetic markers tested),^9^ and so the reported candidate gene associations have been largely discounted.

In 2004, it became feasible to genotype hundreds of thousands of genetic variants in a single experiment.^10^ The development of genotyping arrays enabled the design of genome-wide association studies (GWAS), the first published soon after in 2005^11^. In the years that followed, not only the cost of genotyping arrays decreased substantially, but also methods for the analysis of GWAS data were developed and refined; notably, these included statistical approaches to account for population structure and to infer individual genotypes for genetic variants not present in the genotyping arrays.^12^ As a result, GWAS including data from thousands of individuals genotyped for millions of genetic variants became a reality, at last providing a powerful tool to identify genetic associations with disease risk. For asthma, the first GWAS was published in 2007 by Moffatt *et al.*^13^ Since then, and until the end of 2016, 24 additional GWAS of asthma were published. In this review, we summarize and interpret the key genetic findings from these studies, specifically addressing the following questions: are any published risk variants likely to be false-positive associations? How much variation in disease liability do they explain? What are the likely target genes of those risk variants? Do those genes point to potential new mechanisms underlying disease pathophysiology? Lastly, we conclude by highlighting areas of research that are essential to help translate genetic findings into clinical research or practice.

## Summary of genetic associations reported in asthma GWAS performed between 2007 and 2016

We searched the NHGRI-EBI catalog of published GWAS^14^ to identify studies that tested the association between genetic variants and asthma risk, between 2007 and 2016. We used the search term ‘Asthma’ and applied no filters. The search was performed on the 2nd of August 2017, and returned 73 unique studies, which were individually reviewed for inclusion in our analysis. Of these, 48 (66%) were excluded (Supplementary Table 1), most (34 studies) because the phenotype tested in the GWAS was not asthma, but instead an asthma-related trait (for example, lung function). Studies were also commonly excluded because they were based on DNA pooling (five studies) or reported genetic interactions (for example, gene-by-environment; four studies) rather than main effects. We extracted data from the GWAS Catalog for the remaining 25 studies,^13, 15, 16, 17, 18, 19, 20, 21, 22, 23, 24, 25, 26, 27, 28, 29, 30, 31, 32, 33, 34, 35, 36, 37, 38^ which were included for analysis (Supplementary Table 2).

The definition of asthma was not always the same across the 25 published GWAS. For example, six GWAS ascertained asthma cases with disease onset in childhood, whereas in other GWAS cases had more severe symptoms or co-morbid allergies (Supplementary Table 2). The smallest GWAS included 66 cases and 42 controls,^31^ and the largest 28 399 cases and 128 843 controls.^15^ For most studies (18 of 25), the primary GWAS included exclusively individuals of European descent; two studies were based on populations of Asian ancestry,^27, 29^ two of Latino ancestry,^18, 36^ two of African ancestry^16, 35^ and one included multiple ancestries.^28^ Across these 25 studies, 73 unique genetic variants were reported to associate with disease risk at a genome-wide significance threshold of *P*<5 × 10^−8^ (listed per study in Supplementary Table 3). Some single nucleotide polymorphisms (SNPs) were located in close proximity, and so we used the clump procedure in PLINK^39^ to determine which were likely to represent independent associations and which were simply correlated with previously published variants. Specifically, we assigned each SNP into groups of correlated variants based on pairwise linkage disequilibrium (LD). LD was estimated using data from the 1000 Genomes Project (release 20130502_v5a), separately for individuals of European, Asian and African ancestry, as appropriate. Using a conservative LD threshold of *r*^2^>0.05, there were 31 groups of correlated risk variants reported in GWAS of European ancestry (Table 1 and Supplementary Table 4), with seven, one and three variants reported in GWAS of Asian, African and Latino ancestry, respectively (Supplementary Table 5). One variant reported in Asians (rs1837253) and two (rs9272346 and rs907092) in Latinos were in strong LD (*r*^2^>0.8) with risk variants reported in Europeans, and so do not represent independent associations. Therefore, in total, 39 genetic variants (31 in Europeans and 8 additional in other ancestries) in low LD with each other were reported to associate with asthma risk in GWAS published between 2007 and 2016. In the sections below, we focus on the 31 associations reported in Europeans.

## Are any of the published risk variants for asthma likely to represent false-positive associations?

GWAS, even when conducted using strict quality control procedures and an appropriate genome-wide significance threshold,^40^ are not completely protected from false-positive associations. That is, there is always a small chance that a new genome-wide significant association with asthma risk might not be a true-positive association and instead arise by chance or because of unaccounted methodological biases. As such, whenever possible, it is important to perform an independent and adequately powered replication study to confirm novel associations. This is not always feasible, particularly as GWAS become larger, because there might not be sufficiently large studies available for replication that were not included in the discovery stage.

**Figure 1:**
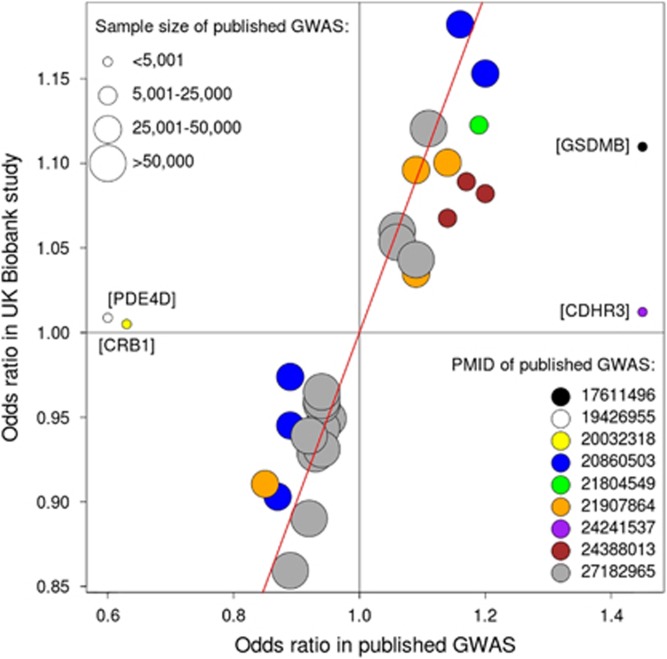
Effect size (odds ratio) for 31 previously reported asthma risk SNPs, comparing results reported in the original GWAS describing each association with those obtained in the analysis of the UK Biobank study. Each SNP is represented by a circle, with its size and color indicating total GWAS sample size (as per Supplementary Table 2) and PubMed identifier (PMID), respectively. The red diagonal line represents equality of odds ratio between published GWAS and UK Biobank (that is, *x*=*y*). The genomic location of the four SNPs that deviate markedly from the diagonal are shown next to the respective circle. For three of these (SNPs in *PDE4D*, *CRB1* and *CDHR3*), the association in the UK Biobank study had a *P*-value>0.05 (see Table 2).

In the last few years, ~500 000 individuals from the UK have been deeply phenotyped and genotyped as part of the UK Biobank study.^41^ The genotype data for the full dataset has just been made publicly available,^42^ providing a unique and timely opportunity to test if the 31 associations with asthma risk reported in Europeans between 2007 and 2016 are reproducible. Briefly, we analyzed data for 380503 unrelated individuals (kinship coefficient indicating <3rd degree relatedness) with (a) European ancestry, confirmed based on analysis of allele sharing with individuals from the 1000 Genomes Project; and (b) non-missing information for field 6152 of the touchscreen questionnaire: ‘Has a doctor ever told you that you have had any of the following conditions?’. A total of 44 003 individuals selected ‘Asthma’ when answering that question, and so were considered as cases. On the other hand, 336 500 individuals did not select ‘Asthma’ and so were considered as controls. Mean age was 56.7 (range 38–72), with 54% of participants being female. We tested the association between individual SNPs and case-control status using SNPTEST, including age, sex and SNP chip as covariates in the model. We adjusted the association results for an LD Score intercept^43^ of 1.073, estimated using 1.2 million HapMap3 SNPs. In this analysis, we were able to test all 31 reported SNPs (all with imputation information >0.98), either directly (30 SNPs) or through a proxy SNP (rs166079 instead of rs200634877, *r*^2^=0.75). Of these, 28 (90%) had a statistically significant (*P*<0.05/31 SNPs=0.0016) and directionally consistent (same predisposing allele as originally reported) association with disease risk (Table 2 and Figure 1), thereby confirming the original findings as true-positive associations.

For the remaining three SNPs, results from this UK Biobank analysis of self-reported doctor-diagnosed asthma did not support an association with disease risk, and so it is possible that they represent false-positive associations. These associations are located in/near *DENND1B/CRB1*,^34^ *PDE4D*^37^ and *CDHR3*^20^. Another explanation for the lack of association with these three SNPs is that the case-control definition we used in the UK Biobank analysis is not a good proxy for that used in the original studies. For example, the original association with rs6967330 in the *CDHR3* gene,^20^ which was subsequently supported by results from Pickrell *et al.*^15^ (rs6959584, *P*=2 × 10^−8^, *r*^2^=0.72 with rs6967330), was found when studying asthma cases with childhood onset and severe exacerbations. If such an association is specific to that subgroup of asthmatics, and if these only represent a small fraction of the UK Biobank asthma cases, then the power to replicate the original association might have been low. In this respect, it is noteworthy that the direction of effect in the UK Biobank for rs6967330 was the same as originally reported. In conclusion, at least 28 of the 31 SNPs previously reported in GWAS of European ancestry have a significant and consistent association with self-reported doctor-diagnosed asthma in the UK Biobank study and so represent *bona fide* asthma risk variants. It was beyond the scope of this review to report associations found in the UK Biobank study that were not located in previously reported asthma risk loci. Studies that report the full results from the UK Biobank study will be reported elsewhere in the near future.

## How much variation in disease liability is explained by the published asthma risk variants and by others yet to be discovered?

Twin studies have estimated the heritability of asthma to be between 55 and 74% in adults,^2, 3^ with even larger estimates reported in young children.^4, 44^ The aim of GWAS is to identify variants that contribute to this heritability. So it is important to understand to what extent the heritability of asthma is explained by the asthma risk variants discovered to date, and how much heritability remains to be discovered. To answer the first question, we estimated the total variance in disease liability explained in the UK Biobank study by each of the 31 published risk variants, using the formula var(*g*)/(var(*g*)* (*π*^2^)/3) described by Pawitan *et al.*^45^ This is often referred to as the SNP heritability. In this formula, var(*g*) for each SNP is given by 2p*(1-*p*)*(log(*OR*))^2^, where *p* and *OR* are respectively the frequency and odds ratio for the effect allele, while *π* is the mathematical constant pi. Using this formula, we found that the median SNP heritability was 0.06% (range 0 to 0.41% Table 2), while the sum of the 31 SNP heritabilities was 2.5%. That is, in the UK Biobank study, 2.5% of the variation in asthma liability is explained by the 31 asthma risk variants discovered to date.

The second question of interest is how much heritability is likely to be explained by risk variants that remain to be discovered. One approach to address this question might be to simply subtract the SNP heritability explained by the 31 published associations (2.5%) from the overall asthma heritability estimates reported in twin studies (*e.g.* 55%). Therefore, potentially, asthma risk variants yet to be discovered could account for at least ~52% (55%–2.5%) of disease liability. However, heritability estimates from twin studies can be inflated, for example, because of violations of study design assumptions.^46^ Thus, such estimates should be taken as the upper boundary of the total variation in disease liability that is explained by genetic variants.

A more conservative approach to address the same question involves first estimating the disease heritability that is explained collectively by all genetic variants studied in a GWAS, not just those with a strong association with disease risk. This is referred to as the SNP-based disease heritability, which can be estimated using for example GCTA,^47^ BOLT-REML^48^ or LD Score regression.^43^ In theory, the SNP-based heritability can be lower than the twin-based heritability if genetic variants not tested (or not well tagged) in GWAS contribute to disease risk, which could be the case for uncommon variants (*e.g.* with minor allele frequency [MAF] <1%). Differences in heritability estimates could also arise if the twin-based heritability estimate is inflated, as discussed above. When we applied the LD Score regression approach to genome-wide results from the UK Biobank GWAS analysis described above (*n*=380 503), we found that the overall SNP-based heritability for asthma was 14%, with a standard error (SE) of 1%. This estimate was obtained based on results for 1.2 million common, well imputed HapMap3 SNPs; therefore, it can be considered as the lower boundary of the total variation in disease liability that is explained by common SNPs. If we consider this estimate, then genetic risk variants yet to be discovered, and that could be identified in larger asthma GWAS of common variants, are likely to account for about 11.5% (14%–2.5%) of the variation in disease liability. This conclusion was supported by the observation that the overall asthma SNP-based heritability obtained after removing from the UK Biobank GWAS the 31 published SNPs (and all variants in LD with them, *r*^2^>0.05) was 12% (SE=1%).

## Have we found fewer asthma risk variants than expected based on GWAS sample size?

The largest asthma GWAS published between 2007 and 2016 included 157 242 individuals and identified 27 independent associations with disease risk. This figure is smaller than reported by GWAS of some complex diseases using similar or smaller sample sizes (Table 3). For example, with a similar sample size (*n*=150 064), Ripke *et al.*^49^ found 128 independent associations with schizophrenia (SCZ), which is 4.7-fold greater than those found for asthma. What underlies this difference in GWAS yield? First, the power to detect an association with a SNP depends on the proportion of variance in disease liability it explains. As discussed above, this can be calculated from the risk allele frequency and the odds ratio. For example, power is about the same to detect an association with two SNPs, one with a risk allele of frequency 0.5 and odds ratio of 1.2, and the other with a risk allele of frequency 0.01 and odds ratio of 2.5—both SNPs explain about 0.5% of variation in disease liability (see formula in section above). When comparing two diseases, one needs to consider that the power to detect an association with a SNP that explains the same proportion of variance in disease liability depends on the disease prevalence.^50, 51^ Using the formula derived by Yang *et al.*,^51^ if we consider a SNP that explains 0.05% of the variation in disease liability, then the expected non-centrality parameter (NCP; which reflects power, but is linearly related to sample size) for the SCZ GWAS listed in Table 3 is 102 (assuming a disease prevalence of 1%). This is 2.6-fold greater than obtained for the similar-sized asthma GWAS (NCP=40, assuming a prevalence of 15%). In other words, although the overall sample size is the same, the SCZ GWAS has substantially greater power to detect an association with such a SNP when compared to the asthma GWAS. Another consideration is the genetic architecture (number of risk loci, their frequency and effect size), which is unknown and may differ across different diseases.^52^ For example, asthma might have a smaller number of common risk variants than SCZ, or SNP effects might be smaller. So to adequately compare the number of associations reported for different diseases (and quantitative traits), one needs to consider not just the sample size but also disease lifetime risk and the likely genetic architecture, as highlighted previously.^50, 53^

Another factor that influences power, and that could have a more severe effect for some diseases than others, is disease heterogeneity and misclassification. If the genetic architecture of asthma is not homogeneous, and instead has components that are specific to clinically distinct subtypes, then lumping all individuals who reported ever having asthma in the same case group could lead to underestimation of SNP heritabilities, which decreases power. As examples of this, the risk allele for susceptibility variants near *ORMDL3* is significantly more common in cases with childhood onset asthma,^6, 54^ while the association near *CDHR3* might be specific to children with early onset and severe exacerbations,^20^ as discussed above. Similarly, including in the control group individuals who do not have asthma but suffer from other common allergic diseases (for example, hay fever) can significantly decrease power to detect associations with SNPs that affect allergies in general, not just asthma.^55^ Shared genetic effects are expected to be widespread amongst common allergic diseases, with pairwise genetic correlations estimated to be >50% in twin studies.^2, 4, 56^ One approach to capitalize on this high genetic correlation is to define cases as those suffering from two or more allergic diseases, and controls as those who do not suffer from any allergic disease.^55^ We have shown empirically that the estimated SNP effects are indeed larger with such approach, and so power is increased.^19^ Another approach that also increases power to detect shared genetic effects is to define cases as those who suffer from any allergic disease.^55^ We have recently performed a large GWAS using this approach (*n*=360 838) and identified 136 independent associations for allergic disease.^57^ Of note, the expected NCP in this study design for a SNP with a 0.05% heritability and assuming a 30% disease prevalence was 123, which is comparable to the SCZ GWAS that identified 128 independent associations.

Lastly, if gene-by-environment interactions have a substantial contribution to asthma risk, then ignoring the relevant environmental exposures in GWAS could also result in underestimated SNP effects. For example, gene-by-environment interactions have been reported for variants in the *ORMDL3* locus, with larger SNP effects found in children with rhinovirus wheezing illness in early life^58^ or exposed to tobacco smoke in early life.^59^ The latter interaction, however, was not replicated in a large independent study.^60^ A small number of genome-wide interaction analyses between environmental exposures and asthma risk have been published,^61, 62, 63, 64^ but are not discussed in this review. For reviews of this topic, see for example.^65, 66^ Thus, in summary, fewer genetic associations have been reported for asthma as compared to some complex diseases with GWAS of similar or smaller size. This likely reflects lower statistical power arising from the relatively high disease prevalence, but also study design limitations, such as not accounting for disease subtypes, information from genetically-correlated diseases and gene-by-environment interaction effects.

## What are the likely target genes of the published asthma risk variants?

The aim of asthma GWAS *per se* is to identify genetic variants associated with disease risk. A genetic variant is associated with asthma risk most likely because that same variant, or another in strong LD with it (for example, *r*^2^>0.8), affects the protein sequence or the transcription patterns of a gene (that is, the ‘target gene’) that plays a role in disease pathophysiology. Therefore, knowing which variants are associated with disease risk might highlight specific genes and molecular pathways dysregulated in asthma, and ultimately help better understand why asthma develops in the first place. How do we identify the likely target genes of risk variants? First, we can determine if the associated variant, or another in strong LD with it, is a non-synonymous coding variant, using for example ANNOVAR.^67^ If we focus on the 28 published risk variants that had a reproducible association with asthma in the UK Biobank study (Table 2), and also include the additional correlated risk variants reported in other asthma GWAS (Supplementary Table 4), then this approach identifies eight likely target genes: *GSDMA*, *GSDMB*, *HLA-DQA1*, *HLA-DQB1*, *IL1RL1*, *IL6R*, *TLR1* and *ZPBP2* (Supplementary Table 6).

A second approach that can be used to identify likely target genes is to determine if the associated SNPs are in strong LD with variants that have been reported to associate with variation in gene expression levels, known as expression quantitative trait loci (eQTL). A plethora of eQTL studies have been reported in recent years, including 39 conducted using tissues relevant to asthma pathophysiology (Supplementary Table 7), for example, whole-blood, lung, skin and individual immune cell types, such as CD4^+^ T cells. For each of these studies, we extracted eQTL results (that is, SNP, gene and *P*-value) from the original publication, keeping only associations in *cis* (that is, SNP within 1  Mb of gene) that were significant at a conservative threshold of *P*<2.3 × 10^−9^, which corresponds to a Bonferroni correction for testing each of 21472 genes^68^ for association with 1000 independent SNPs.^69^ In each study and for each gene, we then used the clump procedure in PLINK to reduce the published list of eQTLs (which typically includes many correlated variants) to the subset of strongest eQTLs that were in low LD with each other (*r*^2^<0.05), which we refer to as sentinel eQTLs. Finally, we asked if any of the 28 variants with a reproducible association with asthma risk in the UK Biobank study (Table 2), or the additional correlated risk variants reported in other asthma GWAS (Supplementary Table 4), were in strong LD (*r*^2^>0.8) with a sentinel eQTL. Using this approach, we found 48 likely target genes (Table 4 and Supplementary Table 8). Of note, these include all eight genes with non-synonymous variants listed above, indicating that for these variation in both protein sequence and transcript levels might be important determinants of cellular function and disease risk.

For many asthma risk variants (12 of 28, or 43%), the two approaches described above failed to identify any likely target gene. This could arise, for example, if the effect of those variants on gene expression is (a) specific to tissues, cell types and/or cellular conditions (e.g., hypoxia) for which eQTL information is not available at present; or (b) too weak to be detected with the sample sizes included in published eQTL studies and at the conservative significance level that we used. In this case, the likely target genes could potentially be identified using functional studies; these can include, for example, experiments to determine the effect of risk variants on promoter-enhancer chromatin interactions, promoter activity or transcription factor binding. We recently used such approaches to identify *PAG1* as a likely target gene of the asthma risk variants located on chromosome 8q21^70^, for which no eQTL support was available at the strict significance threshold used above. In summary, at least 49 genes (including *PAG1*) are likely targets of published asthma risk variants, most (90%) identified based on the LD between risk variants and eQTLs.

## Do any of the likely target genes represent potential new players in the pathophysiology of asthma and allergic disease more generally?

To address this question, we performed a PubMed query using the HGNC-approved gene symbols listed in Table 4, as well as all known aliases (Supplementary Table 9), and the allergy-related terms ‘asthma OR rhinitis OR eczema OR atopic OR dermatitis OR allergy OR allergi* OR hayfever OR 'hay fever'’. We downloaded results from the PubMed query in XML format and then counted the number of unique articles in that file citing both the allergy-related terms and the gene name or aliases in the title, abstract or keyword fields. On the basis of results from this query, we classified the 49 genes into three groups (Table 5). Group one consisted of nine genes co-mentioned frequently (5 or more studies) with those allergy-related terms prior to 2007, the year the first GWAS of any allergy-related trait was published. The genes were *TSLP, IL1RL1, TNFSF4, TLR1, HLA-DQB1, HLA-DQB2, HLA-DQA1, ADORA1* and *TAP2.* For these genes, GWAS findings did not provide the first clue for a key role in disease pathophysiology.

Group two consisted of 13 genes co-mentioned frequently with allergy-related terms since, but not before, 2007: *ORMDL3, GSDMB, ZPBP2, IKZF3, GSDMA, IL6R, CHIT1, FCER1G, SLC22A5, WDR36, IL18RAP, HLA-DQA2* and *NDFIP1.* The first five are located in the same asthma risk locus, and were first suspected to contribute to asthma pathophysiology because of GWAS findings. Of the remaining eight genes, for five it can be argued that functional studies provided the first suggestion of a key role in asthma/allergies, namely *IL6R*,^71^ *CHIT1*,^72^ *FCER1G*,^73^ *IL18RAP*^74^ and *NDFIP1*^75^. But that is unlikely to be the case for the other three genes, *SLC22A5* (organic cation transporter involved in pulmonary absorption of asthma-related drugs^76, 77^), *WDR36* (nucleolar protein involved in processing of 18S rRNA^78^) and *HLA-DQA2* (HLA class II molecule expressed in epidermal Langerhans cells^79^).

Lastly, group three was composed of 27 genes co-mentioned infrequently with allergy-related terms, both before and after 2007. To our knowledge, only two of these genes have been suggested to play a role in allergic disease through functional studies: *USF1*^80^ and *STARD3*^81^. However, many have a known function (Supplementary Table 10) that is directly relevant to allergic disease pathophysiology, such as *F11R*^82, 83^, *MICB*^84^, *CD247*^85, 86^, *PGAP3*^87^, *AAGAB*,^88^ *CAMK4*^89^ and *PEX14*^90^. In summary, of the 49 likely target genes of published asthma risk variants, only 16 were previously implicated by functional studies in disease pathophysiology.

## Are there examples of genetic findings subsequently translated into clinical research or practice?

Based on the Thomson Reuters Cortellis^TM^ Drug database, drugs against five of the 49 likely target genes of asthma risk variants are being considered for clinical development (Table 6). Six of these drugs are being (or have been) tested in clinical trials of asthma. To our knowledge, only one of these clinical studies was motivated directly by results from a GWAS, our clinical trial of tocilizumab (TCZ) in participants with mild to moderate asthma. In 2011, we reported the association between a variant in the *IL6R* gene (rs4129267) and asthma risk.^26^ A consistent association with this variant was later reported also for eczema^91, 92^ and asthma severity.^93^ We and others noted that the disease protective allele (rs4129267:C) was strongly associated with decreased protein levels of the soluble form of the receptor (sIL-6R),^94^ but increased mRNA levels of the full length *IL6R* transcript,^95, 96, 97^ which encodes for the membrane bound form of the receptor (mIL-6R). That is, decreased asthma risk is associated with decreased sIL-6R but increased mIL-6R. By extension, decreased disease risk is likely associated with decreased IL-6 trans-signaling (which requires sIL-6R and is mainly pro-inflammatory) but increased IL-6 classic signaling (which requires mIL-6R and is thought to be mainly regenerative and protective).^98^ On the basis of these genetic findings, it was not immediately obvious what to expect from a drug such as TCZ (approved therapeutic for rheumatoid arthritis and other auto-immune diseases), which blocks both sIL-6R and mIL-6R. A small case study published in 2011 reported decreased clinical activity of atopic dermatitis in three patients treated with TCZ for up to 12 months.^99^ This was the first suggestion of a protective effect of TCZ in allergic conditions. To characterize the effect of TCZ in asthma, in 2013 we performed pre-clinical studies using mouse models of acute allergic asthma.^100^ In these studies, we found that TCZ had a protective effect on allergen-induced airway inflammation only when the experimental model used resulted in increased levels of sIL-6R in the airways, and so that was likely to involve activation of the IL-6 trans-signaling pathway. When that was not the case, dual receptor blockade resulted in worse airway inflammation when compared to control mice. On the basis of the genetic findings and the mouse studies, in 2014 we initiated a clinical trial of tocilizumab in participants with mild asthma, specifically those with CT or TT genotype for rs4129267, as these have markedly increased levels of sIL-6R levels.^94^ Results from this trial are expected to be published in 2018. On the other hand, if our prediction from the genetic studies and findings from the mouse studies are correct, then a drug that blocks sIL-6R but not mIL-6R might be more desirable. There is one such drug (sgp130Fc, also known as FE301 or olamkicept), which was shown to be safe in phase 1 clinical trials^101^ and is now in phase 2 trials for Crohn's disease (Stefan Rose-John, personal communication). Future studies that test the safety and efficacy of this drug in asthma patients are warranted.

## Opportunities and challenges

In this section, we highlight three broad research areas that should be addressed in the short-term to help translate findings from asthma GWAS into clinical research or practice.

### Improving and confirming target gene predictions

As discussed above, eQTL information provides a valuable tool to identify a set of genes for which variation in SNP genotype for an asthma risk SNP is likely to directly cause variation in transcription levels. However, this approach has some caveats, of which we highlight two. First, SNP effects on gene expression can be tissue-specific^102^, context-dependent^103^ and/or relatively small. For these reasons, some genes might not be predicted to be target genes because appropriate (right tissue, context and sample size for that gene) eQTL studies have not been performed. For example, to our knowledge, the largest eQTL study conducted using airway epithelial cells was based on just 105 individuals^104^ (compared to 5311 for whole-blood^105^), and so our understanding of the genetic control of gene expression in this relevant cell type is still very poor. We thus consider essential to continue expanding eQTL studies to include more relevant tissues, diverse cellular stimuli and larger sample sizes. The second caveat of this approach is that a genetic overlap between risk variants and eQTLs can often arise by chance, because eQTLs are widespread.^106^ We therefore argue that eQTL information should be used to generate predictions that can be subsequently tested by functional studies. However, such studies are laborious and time consuming, often deployed one locus at a time (for example, refs. ^70, 107, 108^). As the number of known asthma risk variants increases in the near future, efficient validation of target gene predictions will require high-throughput approaches, such as capture Hi–C,^109^ multiplexed reporter assays^110^ and genome editing.^111^ Ideally, these studies should be performed in relevant primary cell types, which at present is still technically challenging.

### Understanding target gene function and their role in disease pathophysiology

As highlighted above, for many genes it is unclear how variation in gene expression or protein sequence ultimately leads to variation in asthma risk. Therefore, for these, there is an opportunity to make new discoveries regarding the molecular mechanisms underlying the regulation of gene expression, and the impact that this might have on cellular function and disease pathophysiology. The best examples of this to date are *ORMDL3*^112, 113, 114, 115, 116, 117, 118^ and *GSDMB*,^119^ for which recent functional and animal studies have provided the first insights into their contribution to disease pathophysiology. Similarly, we have recently been interested in understanding how variation in *PAG1* expression might contribute to asthma pathophysiology. Our functional studies suggested that decreased *PAG1* expression is associated with decreased disease risk, contrary to what was expected based on the widely accepted contribution of this transmembrane adaptor protein to the development of immune responses.^70^ We are currently testing this possibility using mouse models of experimental asthma. Various models have been described,^120^ including those that mimic mechanisms underlying acute allergic asthma;^121, 122^ chronic asthma;^123^ and viral-induced asthma^124^ in humans. It is not always straightforward to decide which model is most appropriate to use, and different models can result in different findings,^100^ presumably because of the different underlying pathophysiology. In this respect, understanding the contribution of gene expression to cellular function might provide the best clue as to which models to begin with. An additional caveat of these models might be encountered when comparing germline knock-out mice against wild-type mice, because of possible genetic compensation mechanisms in the former, that is, the up-regulation of functionally related genes.^125^ Potential solutions include temporally controlled gene deletion^126^ or using therapeutic agents that block gene activity. Mouse models are also laborious and so are invariably applied on a gene-by-gene basis, which constitutes a significant bottleneck when dealing with the rapidly increasing number of asthma risk genes. Nonetheless, despite the potential limitations of these models, they will continue to represent essential tools to understand the contribution of target genes of risk variants to asthma pathophysiology.

### Genomics-guided prioritization of targets for drug development

GWAS findings, in combination with eQTL information, can provide important clues into the predicted directional effect of gene expression on disease risk. This, in turn, can be used to inform drug repositioning or the development of novel drugs: specifically, if the disease protective allele of an asthma risk variant is associated with decreased (increased) gene expression, then the prediction is that a drug that also decreases (increases) gene expression should similarly have a protective effect on asthma symptoms. For example, the disease-protective allele rs1438673:T^19^ on chromosome 5q22.1 is strongly associated with decreased expression of *TSLP* in skin^127^ (beta=−0.48, *P*=2 × 10^−15^) and whole-blood^103^ (beta=−8.8, *P*=10^−18^). The same direction of effect on gene expression is observed in at least eight different tissues studied by the GTEx project,^127^ including adipose, colon and esophagus. Thus, based on these genetic findings, we can postulate that TSLP antagonists (but not agonists) might improve asthma symptoms. This is consistent with the known contribution of TSLP to disease pathophysiology^128^ and with results from a recent clinical trial of an anti-TSLP antibody.^129^

This is not always so straightforward, as the *IL6R* example discussed above illustrates. Other examples that are harder to interpret occur when the directional effect of a risk variant on gene expression is not the same, or is poorly characterized, in different relevant tissues. For example, the asthma protective allele rs6683383:A is strongly associated with increased expression of *ADORA1* in whole-blood (for example, beta=0.40, *P*=10^−44^ ^130^), suggesting that agonists and not antagonists (such as PBF-680, which is currently being tested in a phase 2 trial in asthma: NCT02635945) might be beneficial in asthma. But it is possible that rs6683383:A has the opposite effect on *ADORA1* expression in other tissues relevant to asthma, such as airway epithelial cells. This example illustrates the importance of characterizing the genetic control of gene expression in multiple relevant tissues and cell types. In this regard, projects such as GTEx^127^ but with a focus on asthma-related tissues and cell types could prove very useful. Tissues and cell types could be selected based on results from tissue-specific expression^131^ or SNP heritability^132^ enrichment analyses. For example, the latter suggests that SNPs associated with allergic disease are enriched amongst enhancers in Th17 cells;^57, 133^ yet, to our knowledge, no eQTL study has been performed on this cell type.

## Concluding remarks

In the last 10 years, GWAS have identified the first reproducible associations between common SNPs and asthma risk. In Europeans, these SNPs account for ~2.5% of the variation in disease liability. The risk SNPs identified provide the starting point for functional studies that can help understand how genetic variation at these loci affects gene expression, cellular function and disease pathophysiology. The target genes of published risk SNPs are likely to include many previously unsuspected players in disease pathophysiology, and so might point to new therapeutic opportunities. Larger GWAS of broadly defined asthma have the potential to identify additional common risk variants that explain at least 12% of disease liability. This could translate into many hundreds or thousands of risk variants, if we consider that the mean SNP heritability is likely to be low (for example, 0.01–0.05%). Larger GWAS will also have increased power to detect individual associations with uncommon variants (for example, MAF<1%), and to quantify their overall contribution to disease liability, which has not been addressed to date. Other areas of research that remain largely unexplored in the field of asthma genetics include understanding if different inflammatory subtypes (for example, neutrophilic and eosinophilic asthma) have distinct genetic components and whether disease remission is a heritable trait. The next decade of research might provide new insights into these important questions.

## Figures and Tables

**Table 1:** Variants in low LD with each other (*r* ^2^<0.05) reported to associate with asthma risk in GWAS conducted between 2007 and 2016 in populations of European ancestry

*Index*	*Chr*	*Bp*	*Context*^a^	*First association reported with P<5 × 10*^−*8*^						*Correlated SNPs reported in other asthma GWAS* b
				*Top SNP*	*Effect allele*	*OR*	*P-value*	*PMID*	*Year*	
1	1	10557251	[PEX14]	rs662064	T	0.94	3.2E-08	27182965	2016	No
2	1	154426264	[IL6R]	rs4129267	T	1.09	2.4E-08	21907864	2011	No
3	1	161159147	PPOX-[]-ADAMTS4	rs4233366	T	1.09	4.8E-15	27182965	2016	No
4	1	167433420	[CD247]	rs1723018	G	0.95	1.4E-08	27182965	2016	No
5	1	173152036	TNFSF18—[]-TNFSF4	rs6691738	T	0.94	2.9E-08	27182965	2016	No
6	1	197325908	[CRB1]	rs2786098	A	0.63	8.6E-09	20032318	2009	No
7	1	203100504	[ADORA1]	rs6683383	T	1.06	1.1E-08	27182965	2016	No
8	2	8458080	[LINC00299]	rs13412757	G	1.06	1.3E-08	27182965	2016	No
9	2	102986222	[IL18R1]	rs3771166	A	0.87	3.4E-09	20860503	2010	Yes
10	2	242698640	[D2HGDH]	rs34290285	G	1.11	1.8E-15	27182965	2016	No
11	3	188402471	[LPP]	rs73196739	T	0.92	6.5E-09	27182965	2016	No
12	4	38799710	[TLR1]	rs4833095	T	1.20	5.0E-12	24388013	2013	Yes
13	5	59369794	[PDE4D]	rs1588265	G	0.60	2.5E-08	19426955	2009	No
14	5	110401872	SLC25A46—[]-TSLP	rs1837253	C	1.19	7.3E-10	21804549	2011	Yes
15	5	131969874	[RAD50]	rs6871536	C	1.14	2.4E-09	21907864	2011	Yes
16	5	141529762	[NDFIP1]	rs200634877	I	0.94	2.5E-08	27182965	2016	No
17	6	31322197	[HLA-B]	rs2428494	T	0.92	1.4E-16	27182965	2016	No
18	6	32728261	HLA-DQA1-[]-HLA-DQB1	rs17843604	T	1.16	1.7E-10	20860503	2010	Yes
19	6	90985198	[BACH2]	rs58521088	T	0.93	7.1E-11	27182965	2016	No
20	7	105658451	[CDHR3]	rs6967330	A	1.45	1.4E-08	24241537	2013	Yes
21	8	81291879	MIR5708—[]—ZBTB10	rs7009110	T	1.14	4.0E-09	24388013	2013	Yes
22	9	6190076	RANBP6—[]-IL33	rs1342326	C	1.20	9.2E-10	20860503	2010	Yes
23	10	9049253	GATA3---[]---SFTA1P	rs12413578	T	0.89	8.1E-12	27182965	2016	No
24	11	76270683	WNT11—[]-LRRC32	rs7130588	G	1.09	1.8E-08	21907864	2011	Yes
25	12	57509055	[STAT6]	rs3001426	T	0.94	1.4E-10	27182965	2016	No
26	14	68749927	[RAD51B]	rs3784099	G	0.94	1.6E-08	27182965	2016	No
27	15	61069988	[RORA]	rs11071559	T	0.85	3.8E-09	21907864	2011	Yes
28	15	67446785	[SMAD3]	rs744910	A	0.89	3.9E-09	20860503	2010	Yes
29	16	11228712	[CLEC16A]	rs62026376	C	1.17	1.0E-08	24388013	2013	Yes
30	17	38069949	[GSDMB]	rs7216389	T	1.45	9.0E-11	17611496	2007	Yes
31	22	37534034	[IL2RB]	rs2284033	A	0.89	1.2E-08	20860503	2010	No

Abbreviations: OR, odds ratio; PMID, PubMed identifier.

a If the top SNP is located within the boundaries of a gene, then the gene name is shown inside square brackets. Otherwise, the two nearest genes (upstream and downstream) are listed, with the distance to each represented by the number of '−' between the square bracket and the gene name.

b SNPs reported in other asthma GWAS and with (1) *r*^2^>0.05 with top SNP and (2) P<5 × 10^−8^ are listed in Supplementary Table 4.

**Table 2:** Association between 31 variants reported in GWAS of European ancestry and self-reported doctor-diagnosed asthma in the UK Biobank study (44 003 cases and 336500 controls)

*Index*	*SNP*	*Chr*	*Bp*	*Context*	*Effect allele*	*MAF*	*OR*	*P-value*	*Asthma h*^*2*^ *explained (%)*
1	rs662064	1	10557251	[PEX14]	C	0.31	1.04	9.7E-006	0.02
2	rs4129267	1	154426264	[IL6R]	T	0.41	1.03	5.8E-006	0.02
3	rs4233366	1	161159147	PPOX-[]-ADAMTS4	T	0.27	1.04	4.7E-007	0.02
4	rs1723018	1	167433420	[CD247]	G	0.41	0.95	5.7E-012	0.04
5	rs6691738	1	173152036	TNFSF18—[]-TNFSF4	G	0.29	1.04	5.8E-007	0.02
6	rs2786098	1	197325908	[CRB1]	G	0.22	1.00	0.5827	0.00
7	rs6683383	1	203100504	[ADORA1]	A	0.33	0.95	5.9E-011	0.04
8	rs13412757	2	8458080	[LINC00299]	A	0.34	0.94	1.8E-013	0.05
9	rs3771166	2	102986222	[IL18R1]	A	0.38	0.90	7.4E-040	0.15
10	rs34290285	2	242698640	[D2HGDH]	A	0.26	0.89	1.5E-039	0.15
11	rs73196739	3	188402471	[LPP]	T	0.17	0.94	4.1E-010	0.03
12	rs4833095	4	38799710	[TLR1]	C	0.21	0.92	1.3E-017	0.06
13	rs1588265	5	59369794	[PDE4D]	G	0.31	1.01	0.2859	0.00
14	rs1837253	5	110401872	SLC25A46—[]-TSLP	C	0.26	1.12	3.4E-041	0.16
15	rs6871536	5	131969874	[RAD50]	C	0.19	1.10	1.0E-024	0.09
16	rs166079	5	141528959	[NDFIP1]	T	0.38	1.04	1.8E-008	0.03
17	rs2428494	6	31322197	[HLA-B]	A	0.47	1.12	6.1E-055	0.20
18	rs17843604	6	32620283	HLA-DQA1-[]-HLA-DQB1	T	0.42	1.18	1.9E-105	0.41
19	rs58521088	6	90985198	[BACH2]	T	0.35	0.93	2.5E-021	0.08
20	rs6967330	7	105658451	[CDHR3]	A	0.17	1.01	0.2233	0.00
21	rs7009110	8	81291879	MIR5708—[]—ZBTB10	C	0.38	0.94	9.6E-018	0.06
22	rs1342326	9	6190076	RANBP6—[]-IL33	C	0.16	1.15	6.4E-048	0.17
23	rs12413578	10	9049253	GATA3---[]---SFTA1P	T	0.11	0.86	1.0E-033	0.13
24	rs7130588	11	76270683	WNT11—[]-LRRC32	G	0.36	1.10	8.9E-033	0.12
25	rs3001426	12	57509055	[STAT6]	C	0.45	1.07	1.4E-021	0.08
26	rs3784099	14	68749927	[RAD51B]	A	0.28	1.06	1.4E-012	0.04
27	rs11071559	15	61069988	[RORA]	T	0.13	0.91	7.3E-017	0.06
28	rs744910	15	67446785	[SMAD3]	A	0.48	0.95	3.5E-014	0.05
29	rs62026376	16	11228712	[CLEC16A]	T	0.25	0.92	7.7E-023	0.08
30	rs7216389	17	38069949	[GSDMB]	T	0.48	1.11	1.7E-044	0.16
31	rs2284033	22	37534034	[IL2RB]	A	0.43	0.97	4.2E-004	0.01
								Total	2.52
								Median	0.06

Abbreviations: MAF, minor allele frequency; OR, odds ratio; *h*^2^: heritability.

Three SNPs with a *P*>0.05 are highlighted in gray.

**Table 3:** Number of associations reported in published GWAS of other polygenic diseases, which were based on a similar or smaller sample size than the largest asthma GWAS published

*Disease*	*N cases*	*N controls*	*N Total*	*N of independent associations in discovery GWAS (P<5x10^−8^)*	*PMID*	*Disease prevalence*	*NCP for a SNP with h*^*2*^=*0.05%*
Atopic dermatitis	18900	84166	103066	21	26482879	20%	24
Asthma	28399	128843	157242	27	27182965	15%	40
Type 2 diabetes	26676	132532	159208	42	28566273	5%	52
Schizophrenia	36989	113075	150064	128	25056061	1%	102
Rheumatoid arthritis	29880	73758	103638	101	24390342	1%	77

Abbreviations: PMID, PubMed identifier; NCP: non-centrality parameter (which reflects power); h2: heritability.

All studies are based on the analysis of 1000 Genome Project SNPs imputed from GWAS arrays (not including fine-mapping arrays).

**Table 4:** Likely target genes of published asthma risk variants identified based on LD with sentinel eQTLs

*Index*	*GWAS SNP*	*Results for sentinel eQTL in strongest LD with GWAS SNP*						*eQTL reported in other studies?* a
		*eQTL*	*LD (r*^*2*^)	*Target gene*	*P-value*	*Study*	*Tissue*	
1	rs662064	rs668805	1.00	*PEX14*	1.2E-019	Zeller	Monocytes	Yes
1	rs662064	rs12028449	0.81	*DFFA*	1.2E-012	Westra	Whole blood	No
2	rs4129267	rs4537545	0.93	*IL6R*	2.0E-029	Westra	Whole blood	Yes
3	rs4233366	rs4233366	1.00	*FCER1G*	5.0E-215	Jansen	Whole blood	Yes
3	rs4233366	rs4233366	1.00	*B4GALT3*	1.5E-026	GTEx	Fibroblasts	No
3	rs4233366	rs4233366	1.00	*ADAMTS4*	1.8E-024	GTEx	Fibroblasts	No
3	rs4233366	rs4233366	1.00	*PPOX*	6.2E-012	GTEx	Fibroblasts	No
3	rs4233366	rs4233366	1.00	*F11R*	3.4E-011	Fehrmann	Whole blood	No
3	rs4233366	rs4233366	1.00	*USF1*	3.4E-011	Fehrmann	Whole blood	Yes
3	rs4233366	rs2070901	0.96	*TOMM40L*	7.3E-010	Naranbhai	Neutrophils	No
4	rs1723018	rs2988279	0.93	*CD247*	3.1E-062	Zhernakova	Whole blood	Yes
5	rs6691738	rs7553711	0.99	*TNFSF4*	1.9E-035	Yao	Whole blood	No
7	rs6683383	rs6683383	1.00	*ADORA1*	6.0E-096	Zeller	Monocytes	Yes
7	rs6683383	rs3766568	1.00	*CHIT1*	6.6E-030	Zhernakova	Whole blood	Yes
7	rs6683383	rs10920570	1.00	*MYBPH*	5.2E-018	Zeller	Monocytes	Yes
7	rs6683383	rs17464408	0.99	*RP11-335O13.7*	8.1E-021	Zhernakova	Whole blood	Yes
7	rs6683383	rs7555556	0.98	*PPFIA4*	8.1E-162	Zhernakova	Whole blood	Yes
9	rs10173081	rs3771180	1.00	*MFSD9*	1.7E-015	Yao	Whole blood	No
9	rs10173081	rs11674302	0.80	*IL18RAP*	7.9E-091	Yao	Whole blood	No
9	rs10173081	rs10189629	0.80	*IL1RL1*	3.0E-012	Zhernakova	Whole blood	No
9	rs3771166	rs11688559	1.00	*AC007278.3*	4.3E-249	Zhernakova	Whole blood	No
12	rs4833095	rs12233670	0.98	*TLR1*	2.8E-057	Battle	Whole blood	Yes
14	rs1438673	rs7723819	0.86	*WDR36*	2.4E-031	Yao	Whole blood	No
14	rs1438673	rs10073816	0.85	*TSLP*	3.3E-012	Zhernakova	Whole blood	Yes
14	rs1438673	rs2289277	0.84	*CTC-551A13.2*	7.0E-029	Zhernakova	Whole blood	Yes
14	rs1438673	rs10051830	0.82	*CAMK4*	1.8E-016	Yao	Whole blood	No
15	rs2244012	rs2246176	0.99	*SLC22A5*	8.7E-014	Westra	Whole blood	No
16	rs166079	rs12655465	1.00	*NDFIP1*	6.8E-115	Zhernakova	Whole blood	Yes
17	rs2428494	rs2428494	1.00	*MICB*	2.3E-011	Walsh	Whole blood	No
18	rs9268516	rs9268400	0.99	*HLA-DRB6*	6.2E-011	Dinarzo	Whole blood	No
18	rs9272346	rs9272346	1.00	*HLA-DQB1*	<4.9E-324	Zeller	Monocytes	Yes
18	rs9272346	rs9272346	1.00	*HLA-DRB5*	2.1E-121	Westra	Whole blood	Yes
18	rs9272346	rs9272346	1.00	*TAP2*	4.1E-011	Westra	Whole blood	No
18	rs9273373	rs1063355	0.99	*HLA-DQA1*	3.6E-154	Raj	Monocytes	Yes
18	rs9273373	rs3134993	0.95	*HLA-DQB1-AS1*	5.6E-058	GTEx	Lung	No
18	rs9273373	rs1063349	0.92	*HLA-DQB2*	1.1E-089	Geuvadis	LCLs	No
18	rs9273373	rs9272545	0.87	*HLA-DQA2*	1.1E-022	Quach	Monocytes	Yes
27	rs10519068	rs11633029	0.86	*RP11-554D20.1*	3.7E-017	Zhernakova	Whole blood	Yes
28	rs56375023	rs17293632	0.98	*AAGAB*	1.7E-013	Zhernakova	Whole blood	Yes
30	rs11078927	rs12946510	0.80	*GSDMA*	<2.2E-016	Hao	Lung	No
30	rs11655198	rs9903250	1.00	*RP11-94L15.2*	5.2E-064	Zhernakova	Whole blood	Yes
30	rs11655198	rs11655198	1.00	*ORMDL3*	7.4E-041	Kasela	CD8 T-cells	Yes
30	rs11655198	rs2305479	0.97	*IKZF3*	5.6E-021	Zhernakova	Whole blood	Yes
30	rs11655198	rs8067378	0.96	*ZPBP2*	2.4E-017	Grundberg	LCLs	Yes
30	rs2271308	rs1053651	0.99	*STARD3*	1.4E-016	Fairfax	Monocytes	Yes
30	rs2271308	rs1053651	0.99	*PGAP3*	9.9E-014	Yao	Whole blood	No
30	rs2271308	rs4795388	0.83	*PPP1R1B*	4.1E-010	Andiappan	Neutrophils	No
30	rs4794820	rs4794820	1.00	*GSDMB*	1.1E-296	Zhernakova	Whole blood	Yes

GWAS SNPs located in the same locus are highlighted in alternating white/gray shading.

a Results from other eQTL studies that provide support for the same target gene are listed in Supplementary Table 8.

**Table 5:** Number of publications co-mentioning gene/alias terms and allergy-related terms

*Gene*	*Prior to 2007*	*Since 2007*
*Group 1: commonly co-cited before 2007*		
* TSLP*	21	814
* IL1RL1*	16	260
* TNFSF4*	9	50
* TLR1*	8	43
* HLA-DQB1*	30	31
* HLA-DQB2*	28	30
* HLA-DQA1*	13	13
* ADORA1*	12	5
* TAP2*	9	2
		
*Group 2: commonly co-cited after 2007*		
* ORMDL3*	0	115
* GSDMB*	0	57
* ZPBP2*	0	18
* IL6R*	2	15
* GSDMA*	0	15
* CHIT1*	2	14
* IKZF3*	0	11
* FCER1G*	1	9
* SLC22A5*	0	7
* WDR36*	0	6
* IL18RAP*	1	5
* HLA-DQA2*	0	5
* NDFIP1*	0	5
		
*Group 3: uncommonly/not co-cited*		
* F11R*	2	3
* HLA-DRB5*	1	2
* MICB*	1	2
* STARD3*	1	2
* PGAP3*	0	2
* PPP1R1B*	0	2
* AAGAB*	2	1
* USF1*	2	1
* ADAMTS4*	0	1
* B4GALT3*	0	1
* DFFA*	0	1
* PAG1*	0	1
* AC007278.3*	0	0
* CAMK4*	0	0
* CD247*	0	0
* CTC-551A13.2*	0	0
* HLA-DQB1-AS1*	0	0
* HLA-DRB6*	0	0
* MFSD9*	0	0
* MYBPH*	0	0
* PEX14*	0	0
* PPFIA4*	0	0
* PPOX*	0	0
* RP11-335O13.7*	0	0
* RP11-554D20.1*	0	0
* RP11-94L15.2*	0	0
* TOMM40L*	0	0

**Table 6:** Drugs against likely target genes of asthma risk variants considered for clinical development

*Gene*	*Drug name*	*Originator company*	*Indications (approved or in trials)*	*Target-based actions*	*Highest status*	*Clinical trials in asthma*
*ADORA1*	Adenoscan	King Pharmaceuticals R&D Inc	Coronary artery disease	Adenosine A1 (and A2) receptor agonist	Launched	NA
*ADORA1*	Trabodenoson	Inotek Pharmaceuticals Corp	Ocular hypertension; Open angle glaucoma; Optic nerve disorder	Adenosine A1 receptor agonist	Phase 3 Clinical	NA
*ADORA1*	Neladenoson bialanate	Bayer AG	Cardiac failure	Adenosine A1 receptor partial agonist	Phase 2 Clinical	NA
*ADORA1*	PBF-680	Palobiofarma SL	Asthma; COPD	Adenosine A1 receptor antagonist	Phase 2 Clinical	NCT02635945
*IKZF3*	Iberdomide	Celgene Corp	Multiple myeloma; Systemic lupus erythematosus	Aiolos inhibitor	Phase 2 Clinical	NA
*IL1RL1*	RG-6149	Amgen Inc	Asthma	IL-33 receptor antagonist	Phase 2 Clinical	NCT01928368
*IL1RL1*	Nerofe	Immune System Key Ltd	Autoimmune disease; Cancer; Diabetes mellitus; Myocardial infarction	IL-33 receptor agonist	Phase 1 Clinical	NA
*IL1RL1*	GSK-3772847	Janssen Research & Development LLC	Asthma	IL-33 receptor antagonist	Phase 2 Clinical	NCT03207243
*IL6R*	Tocilizumab	Chugai Pharmaceutical Co Ltd	Asthma; Chronic lymphocytic leukemia; Motor neurone disease; Rheumatoid arthritis; Scleroderma; and others.	IL-6 receptor antagonist	Launched	ACTRN12614000123640
*IL6R*	Sarilumab	Regeneron Pharmaceuticals Inc	Arthritis; Juvenile rheumatoid arthritis; Rheumatoid arthritis; Uveitis	IL-6 receptor antagonist	Launched	NA
*IL6R*	Siltuximab	Janssen Biotech Inc	Castlemans disease; Multiple myeloma	IL-6 antagonist	Launched	NA
*IL6R*	Sirukumab	Janssen Biotech Inc	Asthma; Major depressive disorder; Polymyalgia rheumatica; Rheumatoid arthritis; Temporal arteritis	IL-6 antagonist	Pre-registration	NCT02794519
*IL6R*	SA-237	Chugai Pharmaceutical Co Ltd	Neuromyelitis optica	IL-6 antagonist	Phase 3 Clinical	NA
*IL6R*	Olamkicept	Conaris Research Institute AG	Inflammatory bowel disease	IL-6/sIL-6R inhibitor	Phase 2 Clinical	NA
*IL6R*	Vobarilizumab	Ablynx NV	Rheumatoid arthritis; Systemic lupus erythematosus	IL-6 receptor antagonist	Phase 2 Clinical	NA
*TSLP*	Tezepelumab	Amgen Inc	Asthma; Atopic dermatitis	TSLP antagonist	Phase 2 Clinical	NCT01405963, NCT02054130
